# Incorporating shared savings programs into primary care: from theory to practice

**DOI:** 10.1186/s12913-015-1250-0

**Published:** 2015-12-30

**Authors:** Arthur P. Hayen, Michael J. van den Berg, Bert R. Meijboom, Jeroen N. Struijs, Gert P. Westert

**Affiliations:** Tilburg School of Social and Behavioral Sciences, dpt. Tranzo (Scientific center for care and welfare), Tilburg University, Address: PO Box 90153, 5000 Tilburg, LE The Netherlands; National Institute for Public Health and the Environment, Centre for Nutrition, Prevention and Health Services, Address: PO Box, 3720, Bilthoven, BA The Netherlands; National Institute for Public Health and the Environment, Centre for Health and Society, Address: PO Box 1, 3720 Bilthoven, BA The Netherlands; Tilburg School of Economics and Management, dpt. CentER (Center for Economic Research), Tilburg University, Address: PO Box 90153, 5000 Tilburg, LE The Netherlands; IQ Healthcare (Scientific Institute for Quality of Healthcare), Radboud University Medical Center, Address: PO Box 9101, 114, 6500 Nijmegen, HB The Netherlands

**Keywords:** Payment reform, Accountable care, Shared savings, Primary care

## Abstract

**Background:**

In several countries, health care policies gear toward strengthening the position of primary care physicians. Primary care physicians are increasingly expected to take accountability for overall spending and quality. Yet traditional models of paying physicians do not provide adequate incentives for taking on this new role. Under a so-called shared savings program physicians are instead incentivized to take accountability for spending and quality, as the program lets them share in cost savings when quality targets are met. We provide a structured approach to designing a shared savings program for primary care, and apply this approach to the design of a shared savings program for a Dutch chain of primary care providers, which is currently being piloted.

**Methods:**

Based on the literature, we defined five building blocks of shared savings models that encompass the definition of the scope of the program, the calculation of health care expenditures, the construction of a savings benchmark, the assessment of savings and the rules and conditions under which savings are shared. We apply insights from a variety of literatures to assess the relative merits of alternative design choices within these building blocks. The shared savings program uses an econometric model of provider expenditures as an input to calculating a casemix-corrected benchmark.

**Results:**

The minimization of risk and uncertainty for both payer and provider is pertinent to the design of a shared savings program. In that respect, the primary care setting provides a number of unique opportunities for achieving cost and quality targets. Accountability can more readily be assumed due to the relatively long-lasting relationships between primary care physicians and patients. A stable population furthermore improves the confidence with which savings can be attributed to changes in population management. Challenges arise from the institutional context. The Dutch health care system has a fragmented structure and providers are typically small in size.

**Conclusion:**

Shared savings programs fit the concept of enhanced primary care. Incorporating a shared savings program into existing payment models could therefore contribute to the financial sustainability of this organizational form.

**Electronic supplementary material:**

The online version of this article (doi:10.1186/s12913-015-1250-0) contains supplementary material, which is available to authorized users.

## Background

Having a strong primary care setting contributes to the functioning of the health care system [1]. Therefore, strengthening the primary care setting is a widely observed policy response to conditions that threaten the sustainability of a health care system. For example, several countries have introduced a gatekeeping system in order to manage the increased demand for specialist services [2], letting primary care physicians control access to secondary care providers.

To date, even countries that are considered to have a relatively strong primary care setting (like the UK and the Netherlands) [1], are looking for ways to address the needs of an ageing and frail population, in which the number of patients with comorbid conditions rises [3, 4]. Government policies have geared towards fostering a whole-patient approach to primary care, meaning that patients are assigned a personal primary care physician who coordinates care across the continuum and substitutes for specialist care when appropriate [5–7].

However, current modes of paying primary care physicians (capitation, fee-for-service, salary) [8] may not be aligned well enough with these policy goals. These payment models neither provide the additional resources necessary for maintaining a whole-patient perspective, nor do they incentivize primary care physicians to act upon their role of managing costs and quality across the continuum of care [9]. They may even run counter to these goals [9, 10]. Although financial micro-incentives (like pay-for-performance) may be used to attain better costs and quality results, a recent survey by the Nuffield Trust suggested that these incentives tend not to stimulate change across the entire continuum of services but rather in narrow clinical areas [11]. Health care professionals proposed to develop risk-sharing arrangements that cover a wide(r) range of services, instead. Also from a theoretical perspective, introducing some form of risk-sharing in which the insurer shares the risk of achieving high costs or suboptimal value with providers, may help to align interests in a setting where interests tend to diverge [12].

Currently a variety of these arrangements are developed and piloted, including shared savings, bundled payment and global payment models [13, 14]. With over 400 participating provider groups and 8 million covered beneficiaries [15], the US Medicare Shared Savings Program receives wide scrutiny. Under a shared savings program payers hold providers accountable for the overall costs and quality of care for a predefined population of patients [16]. Accountability goes beyond reporting about performance to actually share in savings in overall health care expenditures, once quality targets have been met. Providers can reinvest these savings in support of a whole-patient perspective. Furthermore, the prospect of receiving savings in overall expenditures incentivizes to actually deliver the enhanced primary care as envisioned by policy makers. Landon et al. argue that the financial incentives that follow from a shared savings program are best suited to the needs of a strengthened primary care setting, and urge to incorporate shared savings programs directly into enhanced primary care initiatives [9, 17].

In the Netherlands, a pilot with a shared savings program for primary care was introduced for a group of primary care providers (in an additional file we provide a brief description of Dutch primary care, see Additional file 1; primary care providers are hereafter referred to as “(pilot) providers”). This shared saving program included a one-sided payment model, which means that providers only share in savings and not in losses. The program was incorporated in the existing payment model, which is a mix of quarterly capitation fees and fee-for-service. The primary aim of this pilot is to lower spending growth without compromising on the quality of care. The approach is to reorient providers towards value-based care, or to otherwise provide financial support for such an orientation, by introducing the prospect of sharing in expenditure savings, conditional on achieving quality targets.

We describe a structured approach to designing shared savings programs for primary care, discuss pros and cons of alternative design choices, report on our experience in weighing alternative design choices in reaching a final decision, and discuss opportunities and challenges of operating a shared savings program within a managed competition.

### Shared savings programs

**Fig. 1:**
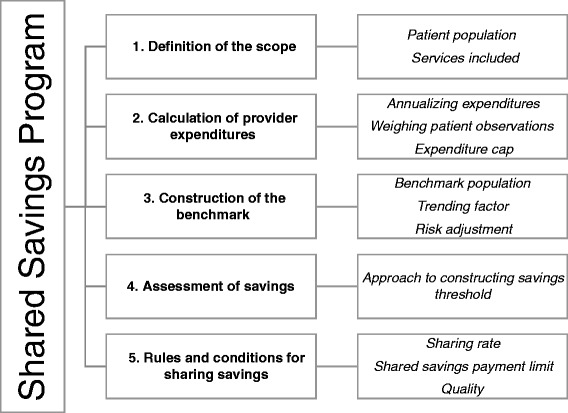
The five building blocks of the shared savings program and their elements. The five building blocks are numbered consecutively from 1 to 5. The blocks in the column on the right depict, for each building block, its elements

We distilled the designs of earlier shared savings models [18–23] into five overarching building blocks of shared savings models, which we use here to ease exposition (Fig. 1): the definition of the scope of the program, the calculation of provider expenditures, construction of the benchmark against which expenditures are assessed, assessment of savings and the rules and conditions for sharing savings. We first provide a background to the building blocks in which we briefly outline the choices to be made. In the remaining sections we describe the pilot program, its shared savings program (in terms of the building blocks) and how it was implemented.

### Risk-sharing under a shared savings program

Under a shared savings program, insurers share part of the risk of increasing expenditures with care providers. For instance, care providers who start investing in their infrastructure in order to generate savings, lose income when the shared savings payments are not enough to cover expenses. Assuming risk-aversiveness on the side of the primary care physicians, uncertainty surrounding the outcome (expenditure savings as determined by the model) is an important driver of behavior. In theory, uncertainty drives investment behavior (choosing low-risk, but low-return investments over high-risk high-return investments) [24], but will also drive up the level of compensation in exchange for which the provider is willing to take on this risk [12]. From this, it follows that risk and uncertainty are important drivers of the program’s efficiency, and that they should be taken into account in the program design.

### Building block 1: Definition of the scope

A first step in designing a shared savings program is to decide on which patients and which health care services to include. These two together define the scope of the shared savings program. Providers only assume accountability for the patients and services under the scope of the program, and only savings made within the scope of the shared savings program count towards shared savings payments.

### Patient population

A common way of defining the patient population is by assigning patients to providers based on some measure of health care usage. The general idea is that providers are held accountable for the overall costs and quality results of patients that have received care from them. One can choose between either prospective (based on a patient’s use of services in the prior year) or retrospective assignment of patients (based on a patient’s use of services in the performance year) [25], and adopt a majority or plurality rule for assigning patients to providers. For example, in the Medicare Shared Savings Program, patients are assigned retrospectively to providers based on where they have received the plurality of primary care services in that year.

### Services included

In general, shared savings programs are targeted at a full set of services, which differs between programs depending on the payer involved and the line of business covered [18]. For example, the Medicare Demonstration Programs and its recent (Medicare) Shared Savings Program include the full set of services furnished under Medicare Parts A and B. The Alternative Quality Contract, launched by Blue Cross Blue Shield Massachusetts, includes all medical services Blue Cross pays for^18^.

Rather than excluding expensive services, one can limit the effects cost outliers have on provider expenditure averages (building block 2) by defining an expenditure threshold. In case a patient’s costs exceed this threshold, the part above the threshold does not count towards provider expenditures. In the Physician Group Practice (PGP) Demonstration Program, expenditures were capped at $100,000. In later versions of the program, expenditures were capped at the 99th percentile of expenditures. For specific subgroups, such as for patients with end-stage renal disease, different expenditure caps were defined such that it created a similar “exposure above group mean expenditures” [19].

### Building block 2: Calculation of provider expenditures

#### Annualizing expenditures, weighing patients observations and the expenditure cap

Provider expenditures are defined as the sum of claims payments made within the scope of the program, including co-payments. Provider expenditures are commonly expressed as average expenditures per year of insurance, which is obtained by annualizing patients’ health care expenditures and calculating a weighted average using patients’ length of enrollment as weights [19, 26]. The cap on expenditures (building block 1) is implemented by truncating annualized expenditures.

### Building block 3: Construction of the benchmark

#### Benchmark population and trending factor

In order to determine whether a provider has realized expenditure savings at the end of the performance year, its expenditures are evaluated against a benchmark. There are several approaches to designing a benchmark. One approach is to design the benchmark such that it can be interpreted as the counterfactual of what health care expenditures would have been had the shared savings program not been implemented. Savings are then defined with respect to historical performance, which is trended forward to the performance year by extrapolating the provider’s growth trend. Such an approach was used in the early years of the Alternative Quality Contract, where this number served as an input for Blue Cross in negotiating budgets with participating Health Maintenance Organizations (HMOs)^18^. Another approach is to design the benchmark such that additional incentives for inefficient providers follow from it. An extreme example of such an approach would be to make the entire benchmark context-independent, by setting it equal to a national or regional expenditure average. In this case, some providers will only outperform the benchmark when they address built-in inefficiency first. Commonly, programs adopt a blended approach. They do use a provider’s historical cost average, but then trend this number forward by a regional (PGP Demonstration) or national (Medicare Shared Savings Program, PGP Transition Demonstration) growth trend [18].

### Risk adjustment

Shared savings programs commonly adjust the benchmark’s base and trending factor for changes in casemix [20, 23, 27]. For those programs adopting a blended approach to designing the benchmark, the historical expenditures that make up the benchmark’s base may reflect a patient population with a case mix different from the provider’s current population. Similarly, the patients assigned to control providers–whose changes in expenditures make up the benchmark’s growth trend–may differ in terms of case mix from those assigned to a participating provider.

Absent a correction for casemix, the composition of a population may play a too large role in determining savings (as determined by the model). The case mix correction needs a careful design, however. First of all, direct standardization of the provider and benchmark population is not suitable for determining a provider’s expenditure savings. Direct standardization gives a provider X’s expenditures had he served the average population, and will therefore not give an indication of realized savings. Rather one wants to calculate the expenditures of an average provider, had he served provider X’s population. Savings are then calculated by subtracting this number from provider X’s realized expenditures. Second, one must decide on the variables to adjust for. An intuitive approach is to add all variables that meaningfully explain variation in individual health care expenditures. This improves the prediction of the counterfactual. The problem with this approach is that some of these variables’ values can be influenced by care providers, e.g. health status. Rather than removing these variables from the risk adjustment model a third choice is to decide on the timing of risk adjustment. This could be either prospective (using information as known prior to the start of the performance year) or concurrent (using performance year information) [28].

### Building block 4: Assessment of savings

#### Approach to constructing the savings threshold

Provider expenditures are evaluated against the benchmark. Random variation in the incidence or progression of illnesses causes random fluctuations in health care expenditures. Accordingly, providers may outperform the benchmark by chance alone but also run the risk that true cost-saving efforts go unrewarded [29]. A general insight from microeconomics is that this kind of randomness lowers the strength of the incentive [30]. Public programs commonly hedge against paying ‘undeserved’ savings [18], by requiring that the difference between benchmark and provider expenditures passes a minimum threshold level. In the PGP Demonstration and the Medicare Shared Savings Program, statistical techniques are applied to determine the confidence intervals on which the savings thresholds are based.

### Building block 5: Rules and conditions for sharing savings

#### Sharing rate and shared savings payment limit

A final set of decisions concerns the sharing of savings between payer and provider. Contracts typically include a sharing rate [18], e.g. that savings will be split evenly between the payer and provider. Public programs like the Medicare Shared Savings Program and the PGP Demonstration added a shared savings payment limit, expressed as a percentage of benchmark spending. Like the savings threshold, the presence of either a sharing rate or performance payment limit serves as a hedge against making undeserved shared savings payments [18]. Other motivations for their presence include the payer’s ability to recoup the losses associated with excess spending [23], and the more general insight from the pay-for-performance literature that the marginal utility from receiving additional income is diminishing [31] (which is an efficiency argument).

#### Quality

As a means to further stimulate value-based provision of health care, accountable care programs often make the net sharing rate dependent on the quality of care [20, 21, 23]. The sharing rate and shared savings payment limit, together determine the amount of savings eligible for sharing; the quality of the care provided determines the amount of shared savings payments made.

The design of this element requires a choice on the dimensions of quality that will be monitored, the operationalization of the quality indicators, scoring, and how these scores will be tied to shared savings payments.

## The Pilot Program

### Structure of the pilot and project members

The pilot includes a large Dutch health insurer and a national chain of primary care providers. The project is led by a steering group, consisting of the general director of the primary care providers and the health insurer’s primary care purchasing manager. The steering group makes decisions on matters related to the project (by consensus) and directs the activities of the project team. The project team’s reports are used as an input for the discussion. This team includes representatives from the health insurer, the care providers involved and the academic research team (the authors). In carrying out its activities, the project group’s members form and chair subcommittees, comprised of employees from a wide variety of departments (depending on the matter at hand, e.g. quality, data management and communication).

### The pilot providers

For evaluation purposes, the decision was made not to include all of the chain’s providers, neither was a random selection of providers deemed appropriate. First, a provider’s population size must be large enough to ensure a satisfactory level of statistical reliability of the cost and quality results [27]. Second, the provider must be capable of assuming the accountability implied by the pilot program. A provider was deemed capable when it is able to (1) routinely collect data on the quality of care, as needed for the calculation of the net sharing rate (building block 5), (2) analyze these and other data such that opportunities for improvement can be identified, (3) create a forum where these opportunities are discussed with its affiliated care providers, and where business plans are made and implemented. Based on these requirements, three providers were selected for the first wave of the pilot. Table 1 provides relevant characteristics of the three pilot providers.

**Table 1:** Characteristics of the pilot PCHMs (December 2014)

	Provider 1	Provider 2	Provider 3
*Dimension*			
Total number of patients (December 2014)	7178	7494	10568
Number of PCPs	5	3	4
PCP compensation	Salary	Capitation fee, fee-for-service, bundled payment, pay-for-performance	Capitation fee, fee-for-service, bundled payment, pay-for-performance
Other care professionals involved	Nurse practitioner (somatic)	(Advanced) nurse practitioner (somatic)	Nurse practitioner (somatic)
	Physician Assistant	Pharmacist	Pharmacist
	Physiotherapist	Physiotherapist	Physiotherapist
Chronic care programs	DM	DM	DM
	COPD	COPD	COPD
	CVRM, secondary	CVRM, secondary	CVRM, primary and secondary
		Osteoporosis	Asthma

### Implementation

The pilot runs from July 2014 to July 2016. For each building block, the research team mapped the full set of decisions that could be made, as well as potential consequences of each choice on statistical reliability and risk. Each building block was discussed in detail with both the health insurer and providers during joint meetings, and their contents were adjusted until consensus was reached. The research team made a number of site visits to explain the model in detail and to present baseline data on health care expenditures and quality. At each site, providers were involved in the choice of investments in infrastructure. Performance with respect to these interventions is a recurring theme during provider meetings. Participating providers also report their results to the steering group, who monitors performance on a quarterly basis. First year results are expected for mid-2016. In the next section, we describe the pilot’s shared savings program. We structure the description of the program by Fig. 1’s building blocks. Each building block starts with the final design choice, followed by a discussion and motivation.

## The Shared Savings Program

### Building block 1: Definition of the scope

#### Patient population

Patients are assigned to a pilot provider only and as long as they (1) take up health insurance from the pilot insurer and (2) are registered with one of its primary care physicians (PCPs). Thus, in case a patient withdraws from the PCP list, the expenditures incurred for medical care received after the withdrawal date no longer count toward a provider’s expenditures under the shared savings program. Regarding (1), we use the health insurer’s administrative database to identify its insured and use its claims database to identify capitation payments, which proxy for the PCP list (2)

Methods of assigning patients to providers have elicited strong debate within US accountable care programs, as concerns were raised about the extent to which prevailing methods yield an accurate reflection of a provider’s patient population [25]. Absent a system of patient registration, patients were assigned on the basis of health care usage and debate has been on whether assignment should be prospective or retrospective. Under prospective assignment, some patients may actually not visit the provider during the performance year for reasons unrelated to health (e.g. when they move out of the area). Under retrospective assignment, not all patients that visit the provider during the performance year may be assigned in the end. Under both methods of patient assignment a provider’s cost-saving intervention may not be fully recouped, because the intervention may either not reach the patient (as could happen under prospective assignment), or because some patients who receive the intervention–and experience a drop in health care expenditures accordingly–will not be assigned to the provider in the end. In case these patients are assigned to benchmark providers instead, the investing provider may actually be harmed by its own investment.

In the Netherlands, all citizens are registered with a PCP [32]. Patients are entitled to (a reimbursement of) non-acute non-incidental PCP care only when they are registered with the providing PCP. It is therefore straightforward to use PCP lists as the basis for assignment, as it is not expected that patients who are registered with a PCP will receive (non-acute non-incidental) PCP care from another provider. Furthermore, Dutch citizens typically maintain a long relationship with their PCP, which makes it easier to manage the population and allows for reaping the long term benefits of improving patient management. A national study [33] found that 60 % has a treatment relationship of over 10 years. Only 9 % has a treatment relationship of less than 2 years. People also tend to be loyal with their health insurer; annual switching rates are low (4-7 %) and about 75 % have not switched insurer since the 2006 health insurance reforms [34]. Thus, a stable patient population can be assumed. Out of the three options, using the GP list as the basis for defining accountability, posed the least risk for participating providers.

### Services included

The scope of the program includes all medical services for which the health insurer provides coverage under both mandatory and supplementary health insurance packages–essentially its full line of business–except dental care services. Patient expenditures count towards provider expenditures up to an amount of €22.500 ($25.376).

Including a large number of services into the shared savings program is congruent with the pivotal role Dutch PCPs play in the health care system. Dental care was excluded from the scope of the program because patients do not need a referral card to visit a dentist. Neither did the pilot providers collaborated with dentists such that accountability could be assumed. Including this type of care thus would have introduced risk that could not have been adequately managed.

The cut-off point of €22.500 protects providers against high health care expenditures that can be ascribed to exceptional individual cases that are beyond the influence of providers. This is desirable: the possibility that random shocks in health and expenditures cause true savings to go unrewarded dilutes incentive strength [30]. The cut-off point was determined jointly with the insurer and provider. In terms of its place in the cumulative distribution of health care costs, our threshold is close to the one used in other programs [20].

### Building block 2: Calculation of provider expenditures

Provider expenditures include both insurer and deductible payments, and are expressed as the person per-annum expenditure average. We first annualize individual expenditures and implement the cut-off point, and then calculate a weighted average over all assigned patients, using assignment length as weight [20]. Formally, annualizing expenditures *E* of a patient *i* assigned to provider *p* in pilot year *t* is done by:

1 $$ A{E}_{ipt}= \min \left({\textsf{C}\hspace{-1.7ex}{=}} 22.500;\ \left(\frac{365}{assignment\  lengt{h}_{ipt}}\right)*{E}_{ipt}\right) $$

And the weighted per-annum expenditure average over all assigned persons is given by:

2 $$ Provider\  Expenditure{s}_{pt}=\frac{{\displaystyle {\sum}_{i\ \in {I}_{pt}}}\left(\left(\frac{assignment\  lengt{h}_{ipt}}{365}\right)*A{E}_{ipt}\right)}{{\displaystyle {\sum}_{i\ \in {I}_{pt}}} assignment\  lengt{h}_{ipt}} $$

Expenditure savings within the domain of a patient’s deductible do not accrue to the health insurer. We nevertheless chose to include deductible payments in the definition of provider expenditures, as a patient’s deductible choice only seems partly related to health and expectations regarding health care consumption [35]. In that case, excluding deductible payments from the calculation of provider expenditures implies that otherwise similar efforts in lowering expenditures would be evaluated differently–depending on the average deductible level across patient populations, which depends on factors unobserved to the research team. Similar to the choice for a cut-off point, leaving out deductible payments would have introduced additional risk that could not have been managed by the care providers.

### Building block 3: Construction of the benchmark

#### Benchmark population and trending factor

A provider’s expenditure savings are evaluated against a benchmark. The benchmark consists of two parts. The first part is the provider’s historical pre-pilot three-year weighted average of provider expenditures *WPA*_*p*_, with larger weights attached to more recent years (0.1; 0.3; 0.6 respectively) [20]. We refer to this part as the ‘base’ of the benchmark, and we denote the three base years by ‘base year (BY) 1,2 and 3’ with base year 3 being the most recent year. This is the year prior to the start of the pilot (baseline; ‶0″ in subscripts). The second part of the benchmark is the growth in provider expenditures from baseline to the performance year of interest 1 + *g*_*ct*_, for a control group of randomly sampled non-participating providers (N = 50) in the region surrounding the pilot area.

Formally, the benchmark for a pilot provider *p* in pilot year *y* = *t* is denoted by:

3 $$ Benchmar{k}_{pt}=WP{A}_p*\left(1+{\mathit{\mathsf{g}}}_{c,t}\right) $$

In which

4 $$ {\mathsf{g}}_{\left(c,t\right)}={\varPi}_{\left(y=1\right)}^t\left(1+{\mathsf{g}}_{cy}\right),\mathrm{with}{g}_{,cy}=\frac{Provider\  expenditures\ \left(c,y\right)- provider\  expenditures\ \left(c,y-1\right)}{provider\  expenditures\ \left(c,y-1\right)} $$

In the implementation section we describe how the parameters of the benchmark are obtained.

The historical expenditures that together form the base of the benchmark create a ‘starting position’ that should lie within reach for pilot providers. Additional incentives for cost containment for inefficient providers follow from making the growth rate context-independent. Research on goal setting theory suggests that performance increases in goal difficulty, up to the point where the difficulty of the goal lies outside one’s ability [36]. Following this research, it is expected that a combination of both approaches yields better performance (especially for low performers) than when the benchmark is shaped according to one approach only: outperforming the counterfactual might prove too easy, whereas a benchmark that does not take into account the particularities of the respective provider (e.g. its population), may not be within the provider’s reach.

### Risk adjustment

The expenditures of base year 1 and 2 are expressed in terms of base year 3′s case mix. By doing so, both numbers will reflect what historical expenditures would have been had the patient population been similar to the population at *baseline*. On top of that, these numbers are trended forward to baseline euros (€) to account for inflation and other periodic effects (e.g. changes in regulation like an expansion of the universal mandatory health insurance package). Similarly, the growth trend is adjusted for case mix differences between control providers and the pilot provider, such that they reflect what the growth in health care expenditures would have been, had the control group witnessed a change in case mix similar to that observed in the pilot provider.

We adjust the benchmark for case mix differences in demographics, socioeconomic status and health, and periodic effects (further explained in the implementation section below), as they have explained variation in health care expenditures between providers before [37]. We adjust for differences in demographics and socioeconomic status on a concurrent base, and prospectively adjust for differences in health [20]. Adjusting on a prospective base means that we set its value equal to its level upon assignment and hold it fixed for the assignment period. In an additional file we provide an overview of all variables and how they are adjusted for (see Additional file 2).

Concurrently adjusting for health status implies that care providers could inflate their benchmarks by upcoding diagnoses [20]. Furthermore, concurrent adjustment for health status lowers the incentive to prevent illness, in particular those conditions that are well-compensated. As a means to enhance incentives for prevention and cure, we therefore adjusted for health status on a prospective base and use concurrent risk-adjustment for all other variables.

### Building block 4: Assessment of savings

#### Approach to constructing the savings threshold

The level of provider savings is determined by evaluating provider expenditures against the benchmark. We use statistical hypothesis testing to determine whether any observed difference in provider and benchmark expenditures is large enough to rule out randomness with statistical confidence. The relevant hypothesis test is a one-sided t-test for ‘no savings or losses’ (the null hypothesis) versus ‘savings’ (the alternative hypothesis) [27]. Under the statistical test, we need to determine a threshold value (the alpha) above which we reject the null hypothesis of no savings. This threshold has important implications for the pilot providers as it determines whether ‘savings’ are labeled as such; a stricter threshold value protects the payer from making undeserved shared savings payments, but it also increases the type-II error of not rewarding true savings–the prospect of which dilutes incentives [30]. Thus, rather than using threshold values from other accountable care programs (commonly expressed as a percentage of benchmark expenditures) or sticking to “conventional levels of statistical significance”, Pope and Kautter [27] advocate a more pragmatic analysis of setting the threshold, based on Power Analysis. We follow this approach and determine the exact values upon receiving the baseline data.

### Building block 5: Rules and conditions for sharing savings

#### Sharing rate and shared savings payment limit

The health insurer and pilot providers agreed on a sharing rate and a shared savings payment limit, which are not reported here for reasons of confidentiality. The payment limit is based on a percentage of provider revenue.

### Quality

For the first wave of the pilot, quality is assessed on four domains: patient satisfaction, chronic care, drug prescription behavior and practice management. These domains were determined jointly with the pilot insurer and providers, based on beliefs of providers’ current span of accountability, data availability and reliability. The resulting domains predominantly reflect health care services that are delivered or coordinated by the pilot providers themselves. Nevertheless, these domains may be changed in later waves to include more specialist services, should pilot providers start engaging in formal contractual arrangements with specialist providers.

**Fig. 2:**
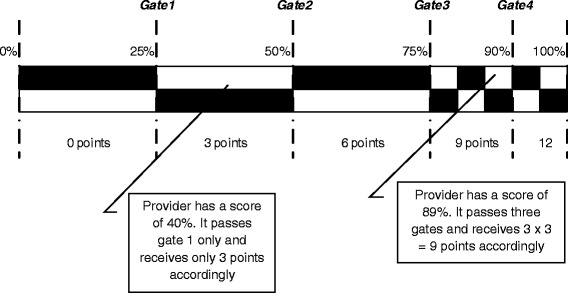
Earning points for absolute performance. The black and white checkered bar represents the score obtained on a specific indicator, ranging from 0 % to 100 % of the maximum achievable score. The bar is subdivided into five gates. For each gate passed, a provider receives three points

Within each domain, quality indicators are formulated and scored on a 0-100 % scale such that a 0 % score and a 100 % score reflect ‘worst’ and ‘best’ possible performance respectively. Performance on these scales is measured in absolute terms. Performance scales are subdivided into gates [21], and in passing a gate a provider receives 3 performance points for absolute performance. Figure 2 visualizes this pay-for-performance element. In an additional file we provide an overview of all quality indicators (see Additional file 3).

**Fig. 3:**
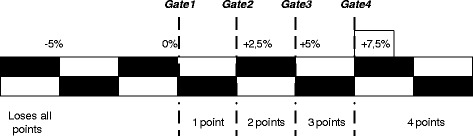
Earning points for improving year-to-year performance. *Earning points for improving year*-*to*-*year performance*. The black and white checkered bar represents the score obtained for improvement on a specific indicator, ranging from–5 % to +7.5 % (and up). The bar is subdivided into four gates. For each gate passed, a provider receives one point

On top of earning performance points, providers can also earn points for improving their performance relative to last year. This has a similar system (Fig. 3):

Providers earn 1 point for passing a gate (implying a 1:3 ratio for improvement and absolute performance [38]). In case a provider’s performance has declined more than 5 % over the performance year, it receives no points for performance improvement, and loses all its points earned for absolute performance. This should serve as a penalty for providers who try to lower expenditures by lowering quality. As scores change from year to year depending on population characteristics, we chose not to impose this penalty for declines smaller than 5 % already. Ultimately, the percentage of savings shared depends on the provider’s overall quality score. The overall quality score is obtained by first summing performance and improvement points over all indicators, by domain, and dividing it over the maximum score (16 per indicator) to obtain a domain average. Second, these domain averages are summed and averaged to obtain an overall quality score. In case a provider scores 60 %, it will receive 60 % of the sharing rate of savings, which, in case of a 50 % sharing rate, is 30 % of overall savings.

Eijkenaar [31] provides an overview of the contributions done in the area of the design of quality payment programs. The incentives following from the “quality payment program” in the shared savings program (i.e. the savings a provider receives are related to the quality of the care provided), are targeted at the group (provider) level rather than at the level of the individual physician. According to Eijkenaar [31], this is preferred in case improvement needs collective action, which we believe holds in our setting where a multitude of disciplines cooperates in the treatment of a wide range of patients, and where quality is measured on dimensions related to, among others, chronic care and patient satisfaction. Moreover, pilot providers indicated that they refer to colleagues when these are known to have particular expertise in the field (e.g. dermatology).

The performance goals are absolute rather than relative, to foster collaboration and sharing best practices with one another [21]–the pilot providers operate in each other’s vicinity. We define multiple targets (i.e. gates) and reward for both absolute performance and improvement such that, in principle, low performers are also incentivized [31]. In later waves of the pilot, the level of the lower and upper targets may be adjusted up or downwards depending on the state of affairs, similar to what was done in the Alternative Quality Contract (data to do so already were not available on a national scale when the program was designed).

## Methods

### Obtaining the benchmark’s parameters

We start with describing our approach to obtaining the growth rate ${\mathit{\mathsf{g}}}_{ct}$ first. To obtain this rate, we estimate a model of the log of provider expenditures (see expression 2). In our model we include case mix variables, year dummies and an interaction between the year dummies and the control providers *p* = *c*. The case mix variables are operationalized as proportions, expressed in assignment days (e.g. “the sum of assignment length over all women in provider *p*’s population, divided over the sum of assignment days). That is, even though a provider may have an equal number of men and women assigned to it, the average assignment length ultimately determines the reported proportion. Formally, the model is described by:

5 $$ Ln\left( Provider\  Expenditure{s}_{pt}\right) = \mu +{\varXi}_{pt}^{\hbox{'}\ }B+{D^{\hbox{'}}}_{year}(t)\varGamma +\left[{D}_{year}(t) \times {C}_p\right]D+{\alpha}_p+{\epsilon}_{pt} $$

In which Ξ_*pt*_ is an *m* × 1 column vector of *m* demographic, health (care) and socioeconomic variables (Additional file 2), *D*_*year*_ are year dummies and *C*_*p*_ is an indicator variable for whether a provider belongs to the set of 50 providers that together form the control group of randomly sampled non-participating providers (N = 50). *B*, Γ and *D* denote the accompanying parameter vectors. Their elements are denoted by small-case characters β_*m*_, γ_*t*_ and δ_*t*_ respectively. *α*_*p*_ ~ *IID*(0, *σ*_*a*_^2^) and *ϵ*_*pt*_ ~ *IID*(0, *σ*_*ϵ*_^2^) together form the model’s error term, with α_*p*_ representing the provider specific component and ∈_*pt*_ representing the remainder component.

Due to the log-transformation of provider expenditures, the parameters can be interpreted as semi-elasticities (i.e. they inform about the percentage change in provider expenditures in response to a change in *B*, Γ or *D*). This is a convenient property for calculating the rate of growth in expenditures of the control group over two consecutive years *t* − 1 and *t*, as it equals the sum of (γ_*t*_ − γ_*t*-1_) and (δ_*t*_ − δ_*t*-1_) under a constant case mix. To predict what this rate of growth had been had the control group witnessed a change in case mix similar to that observed in the pilot provider *p*, we must impute pilot provider *p*’s values for Ξ_*t*_ and Ξ_*t*-1_. Formally:

6 $$ {\tilde{\mathit{\mathsf{g}}}}_{\left(c,y\right)}= \ln \left( Provider\; Expenditure{s}_{ct}\right)- \ln \left( Provider\; Expenditure{s}_{ct-1}\right)=\left({\varXi}_{pt}^{\prime }-{\varXi}_{pt-1}^{\prime}\right)B+\left({\gamma}_t-{\gamma}_{t-1}\right)+\left({\delta}_t-{\delta}_{t-1}\right) $$

(Note that ${\tilde{\mathit{\mathsf{g}}}}_{\left(c,y\right)}$ is formally modeled as the rate of growth of the pilot provider *p* plus any additional growth in the control group.)

The base of the benchmark is a weighted average of three years of pre-pilot spending. The most recent year can be considered the baseline year. A first step in arriving at this weighted average is to first calculate provider expenditures as under (2), for each base year. A second step is now to express base years’ 1 and 2 expenditures in terms of the case mix at baseline [39]. Due to the log-transformation of provider expenditures, the parameter estimates in *B* and Γ, can be used to estimate the percentage change in provider expenditures as a response to either changes in case mix and periodic effects pertaining to the provider (inflation). For example, the parameter estimate β_*women*_ informs about the percentage change in average provider expenditures in response to a change in the proportion of women. By subtracting the proportion of women in provider *p* in base year 1 from that of base year 3, and subsequently multiplying this number by β_*women*_, one can estimate the change in provider expenditures for base year 1, had the provider witnessed a similar proportion of women back then. When we do this for all elements in *B* and Γ (for the particular years) we arrive at the total percentage change in provider expenditures. A third step is to then multiply base years’ 1 and 2 expenditures by the summation of 1 plus this percentage change (e.g. this totals 1,05 for an estimated change in expenditures of 5 %). The final steps are to multiply the resulting number by the weights assigned to each year (0,1 for base year 1; 0,3 for base year 2 and 0,6 for base year 3) [20] and to sum over each base line year to determine *WPA*_*p*_.

### Structural support

Accountable care programs aim to incentivize providers to alter their behavior, in particular those behaviors associated with managing a patient population. Realizing savings may thus require changing complex and entrenched behaviors. Furthermore, the shared savings payments to pilot providers are determined over a full year of care provision and over a care continuum which extends well beyond the providers’ own setting.

Systematic reviews of the effectiveness of pay-for-performance programs suggest that these programs are most effective at changing “simple, discrete and time-limited ” behaviors [40], which, by their nature, may not be the behaviors that lead to significant or enduring savings by the end of the performance year. When targeted at complex and entrenched behaviors, pay-for-performance programs seem most effective when accompanied by supportive strategies [40]. During the pilot, providers receive support in a number of respects. For example, it was recognized that providers have incomplete data on their patients’ health care use and costs. This holds in particular for care delivered within other segments (such as specialist care). The pilot providers indicated that this prevented them from obtaining an adequate picture of their population’s health care consumption, which was deemed an essential input for redesigning population management. Complementing these data with appropriate benchmarks gave hints for improvement.

The pilot providers were asked to indicate their data needs. Generally, these data were available at the health insurer (e.g. most expensive specialisms, hospital treatments, laboratory tests at the population level, health care usage of the most expensive patients). Benchmark data on hospital care (e.g. number of treatments per specialism and types of treatment per specialism (inpatient, outpatient, same day admission/discharge)) were obtained from the Dutch National Atlas of Public Health [41]. Other benchmark data will be provided upon receiving data of the population of patient assigned to providers in the control group. The data analysts of the insurer, the pilot providers and the academic team were involved to ensure correct interpretation of the information. This process will repeated throughout the pilot, should data needs change.

## Discussion

### Pilot aims

The overall aim of implementing the shared savings program is to lower spending growth without compromising on the quality of care. The approach is to reorient providers towards value-based care, or to otherwise support such an orientation by adapting the payment system along this line. The accountable care program and supportive strategies aid may strengthen primary care providers in redesigning the structures and processes of health care delivery. Extensive data sharing allows providers to identify the inefficiencies built up in the continuum of care; the prospect of shared savings payments incentivizes providers to address these inefficiencies. As shared savings payments are determined with respect to the care continuum, pilot providers are furthermore motivated to change their configuration to one that supports substitution of specialist care, coordination across silos, prevention and self-management.

### Pilot opportunities

The pilot system provides a number of opportunities for achieving the pilot aims that are typically not found in other initiatives. First, Dutch citizens enroll with a PCP and their enrollment is typically long-lasting, which facilitates the design of interventions targeted at achieving the pilot aims, and rewards adopting a long-term perspective.

Second and related, since the method of assigning patients to pilot providers is not based on actual health care usage but on enrollment instead, less patients will be “lost to follow-up” [42], because also the scores on quality indicators for patients that do not (regularly) visit the provider count towards the provider’s average. In contrast, patients that do not visit the provider except in case of emergency will not be assigned to the provider under the Medicare Shared Savings Program, because emergency care is not part of the definition of ‘primary care services’ upon which assignment is based [42]. Including these (generally vulnerable) patients in the program incentives providers to also reach out for them, involve them in tailor-made care programs, and to improve care accordingly [42]. However, an assignment system based on registration could provide incentives for risk selection: providers could deny access to high risk patients, or may take actions that lead to an outflow of high risk patients to neighboring providers. This will be a topic of further study.

A third opportunity is that the shared savings payments are at the provider level (and not on the level of the individual PCP), which encourages collaboration within pilot centers and recognizes that potential successful interventions are group efforts. A US study on accountability relationships within participating providers found that rewards were at the level of the individual provider despite recognizing that program aims would be more readily achieved by group effort (i.e. collaboration between individual providers) [43]. Similarly, quality is scored in absolute terms rather than in relative terms, to encourage collaboration between providers.

A fourth opportunity arises from the small scale of the pilot. Due to this small scale, it is not computationally burdensome to calculate savings thresholds for each pilot provider separately. In larger initiatives, predetermined thresholds tend to be used^8^. Hypothesis testing provides a more accurate way of addressing the random variation in health care expenditures and the odds of outperforming the benchmark by chance.

### Pilot challenges

The pilot system faces a number of challenges in achieving its aims. One challenge comes from the fragmented structure of the Dutch health care system. Whereas in the US a substantial number of accountable care providers include a hospital [26], the functions of primary and secondary care remain rather separate in the Netherlands. It is unsure whether pilot providers, in their attempts to lower health care expenditures, succeed in involving specialist providers–in particular because specialist payment models have a volume component. Earlier experiments in accountable care programs targeted at primary care providers, resulted in an increase in expenditures of secondary care. Although it is yet unclear why such a trend occurred, one hypothesis is that specialist providers make up for a drop in the patient volumes (−25 % compared to care-as-usual under the experiment), by increasing treatment intensity (+ €142) [44].

A second challenge is that specialist care is billed retrospectively. Specialist care is classified into packages called ‘diagnosis-treatment combinations (DTC)’, which have a duration of 1 year. Only upon billing the DTC, health insurers know that a particular individual received specialist care. For those DTCs with a starting date of late 2014, the billing date is expected to be mid-2016. The late billing time for specialist care implies that shared savings payments can be determined 1 to 1,5 years after the end of the performance year. A first implication is that the incentives will be less strong for this delayed lump-sum as people tend to discount future payments [45]. It furthermore adds uncertainty [30]. As of 2015, the duration of DTCs will be drastically shortened to 120 days however, which speeds up this process. This also allows the health insurer to provide interim insight on specialist care use for pilot providers. The pilot providers standardized the process of registering patient referrals so that information on secondary care use will become available earlier.

### Experience with GP fundholding

Several national health care systems have gained experience with holding care providers accountable for the costs and quality of health care. An interesting case in this respect is the English National Health Service, because its programs were targeted at GPs as well. Furthermore, NHS GPs are considered gatekeepers too and have worked separately from hospitals. Several programs and reforms have been implemented over the years, among which are the Quality and Outcomes Framework [46], Clinical Commissioning Groups [47] and GP Fundholding [48]. The latter bears most resemblance to the Shared Savings Program. Under GP Fundholding (1991–1997), GPs received a budget to commission health services on behalf of their population, and were entitled to the savings against this budget. Although there are only a few empirical studies of good quality on this topic [49], research suggests that the *abolition* of this fundholding scheme led to an increase in admission rates for chargeable elective admissions of between 3,5 – 5,1 % [50]. Research also documents a lower growth rate in prescribing costs in fundholding practices [51]. The evaluation of the Shared Savings Program will ultimately show whether pilot GPs are able to exert similar influence over the value of care delivered across the continuum. However, there are a number of fundamental differences between both programs that could change the Shared Savings Program’s relative performance. First and foremost, the pilot GPs are not tasked with commissioning health services of behalf of their patients, which is still done by the health insurer. Thus, pilot GPs may have a somewhat less direct influence on where patients will go for treatment. Second, the Shared Savings Program’s benchmark against which savings are calculated is determined ex post, rather than ex ante. Although this could yield a more precise estimate of true savings, this does increase uncertainty over the size of the shared savings payments which lowers the strength of the savings incentive. Third, the benchmark in the Shared Savings Program is more advanced in terms of case mix correction (under GP Fundholding, the budget was determined based on historical expenditures). This, in turn, makes it easier to identify and reward true savings efforts, which lowers uncertainty. Fourth, in the Shared Savings Program, a link is established between the level of shared savings payments and the quality of care, which could lower the incentive to skimp on quality or to stint on care to save costs. In contrast, under GP Fundholding, research documented a drop in patient satisfaction [52], which is explicitly monitored and rewarded here. Whether these differences in design (or institutional environment) drive any difference in the relative performance of both models is a topic of further study.

### Research plan

The impact of the Shared Savings Program will be evaluated on three dimensions: quality, accessibility and expenditures. Since we did not have an opportunity to collect similar quality data for the control group, changes in quality will be assessed using before-after studies (including the indicators that cover accessibility). In terms of expenditures, we are able to use difference-in-difference designs commonly used to evaluate Shared Savings Models [53, 54]. Propensity scores can be used to account for selection-into-treatment [55]. Apart from this general evaluation, we will evaluate several design choices in terms of their statistical properties. These include the choice for a cut-off point in health care expenditures, the threshold value in the savings test, and the performance of the risk-adjustment model. As for the latter, since the method of assigning patients to pilot providers is based on active registration, we will simulate the Program’s results under different scenarios of risk selection to check whether this would be a viable strategy for participating providers.

## Conclusion

Shared savings programs are a relatively new phenomenon and it remains to be studied whether prevailing designs are optimal. This papers demonstrates that a common theme appears to be the minimization of risk and uncertainty, in the presence of which providers will be discouraged to invest in a whole-system perspective. Health system characteristics play an important role here. Dutch PCPs assist patients in navigating the health care system. This allows for a broader scope of the shared savings program, in the sense that few parts of the care continuum are a black box to the accountable provider. However, due to institutional fragmentation within the Dutch health care system, Dutch providers are typically small in scale and scope, which could pose challenges to the viability of the shared savings programs. Savings might be offset by providers not incentivized by the contract and, under prevailing (statistical) minimum savings thresholds, the efforts required to gain from participating in the program may be large. These will be important topics for evaluation.

Irrespective of these challenges, incorporating a shared savings program does reward taking accountability for overall costs and quality results, and could thereby improve the sustainability of the primary care setting. Furthermore, since patients typically have a long-lasting relationship with their personal physician, accountability can more readily be assumed and monitored properly; if one is able to condition on a relatively stable population, the confidence with which changes in costs and quality can be related to changes in population management (rather than to changes in the composition of the patient population), improves. Thus, shared savings programs fit particularly well into the concept of enhanced primary care. Testing this principle for a non-US health care system–like under the current pilot–increases our understanding of shared savings programs, in particular of the mechanisms through which incentivized providers can control spending growth and improve quality.

## Additional files

Additional file 1:
**A brief description of the Dutch primary care sector.** Provides important information of the Dutch primary care sector (DOCX 11 kb) Additional file 2:
**Casemix variables and method of adjustment.** Lists all the variables we use for casemix adjustment, and whether these are adjusted for on a prospective or retrospective base (DOCX 14 kb) Additional file 3:
**Quality indicators. Lists all the quality indicators and their source.** (DOCX 14 kb)

## Abbreviations

DTC: diagnosis-treatment combination
HMO: health maintenance organization
PC: postal code
PCP: primary care physicians
