# Supplementary material for: Incorporating shared savings programs into primary care: from theory to practice
Source: BMC Health Serv Res. 2015 Dec 30;15:580. doi: 10.1186/s12913-015-1250-0 (PMC4696086; doi:10.1186/s12913-015-1250-0)
Supplement: Additional file 1: — A brief description of the Dutch primary care sector. Provides important information of the Dutch primary care sector (DOCX 11 kb) [file 12913_2015_1250_MOESM1_ESM.docx]

**A brief description of the Dutch primary care sector**

In the Netherlands, each patient has its own personal primary care physician (PCP), who serves as a patient’s first point of contact with the medical system. The PCPs in the pilot and the control group commonly lead a multidisciplinary team of care providers (e.g. dietician, physiotherapist, mental health worker), arrange care with other professionals and coordinates and integrate the provision of chronic care. PCPs also provide preventive care (e.g. flu shots), acute and curative, and palliative care. Dutch PCPs offer enhanced access to care (evening consultations, access to out-of-hours PCP cooperatives, non-visit-based services). These services are reflected in the fee schedule.
