# Supplementary material for: Incorporating shared savings programs into primary care: from theory to practice
Source: BMC Health Serv Res. 2015 Dec 30;15:580. doi: 10.1186/s12913-015-1250-0 (PMC4696086; doi:10.1186/s12913-015-1250-0)
Supplement: Additional file 2: — Casemix variables and method of adjustment. Lists all the variables we use for casemix adjustment, and whether these are adjusted for on a prospective or retrospective base (DOCX 14 kb) [file 12913_2015_1250_MOESM2_ESM.docx]

**Casemix variables and method of adjustment**

| ***Variable*** | ***Source*** | ***Type of risk-adjustment*** |
| --- | --- | --- |
| **Age** | Health insurer’s administrative files | Concurrent |
| **Died y/n** | Health insurer’s administrative files | Concurrent |
| **Gender** | Health insurer’s administrative files | Concurrent |
| **4 digit postal code (pc)** | Health insurer’s administrative files | Concurrent |
| **Urbanity 6-digit PC** | Health insurer’s administrative files / Statistics Netherlands [1] (data records were linked by a Trusted Third Party) | Concurrent |
| **% Non-western immigrants 6-digit PC** | Health insurer’s administrative files / Statistics Netherlands [1] (data records were linked by a Trusted Third Party) | Concurrent |
| **Household composition 6-digit PC (several variables)** | Health insurer’s administrative files / Statistics Netherlands [1] (data records were linked by a Trusted Third Party) | Concurrent |
| **Average gross monthly income 6-digit PC** | Health insurer’s administrative files / Statistics Netherlands [1] (data records were linked by a Trusted Third Party) | Concurrent |
| **Socio economic status score 4-digit PC** | The Netherlands Institute for Social Research [2] | Concurrent |
| **Pharmacy cost group (as defined by the National Health Care Institute)[3]** | Health insurer’s claims files / Koninklijke Nederlandse Maatschappij ter bevordering der Pharmacy | Prospective |
| **Diagnosis cost group (as defined by the National Health Care Institute)[3]** | Health insurer’s claims files | Prospective |

**References**

1. Statistics Netherlands. Kerncijfers Postcodegebieden 2008-2010. 2012.

2. The Netherlands Institute for Social Research. Statusscores 1998-2010. 2012.

3. The National Health Care Institute. 2015. <http://www.zorginstituutnederland.nl/>.
