# Supplementary material for: Incorporating shared savings programs into primary care: from theory to practice
Source: BMC Health Serv Res. 2015 Dec 30;15:580. doi: 10.1186/s12913-015-1250-0 (PMC4696086; doi:10.1186/s12913-015-1250-0)
Supplement: Additional file 3: — Quality indicators. Lists all the quality indicators and their source. (DOCX 14 kb) [file 12913_2015_1250_MOESM3_ESM.docx]

**Quality indicators**

| ***Patient reported quality of care (global scores 0-10)**** |
| --- |
| The ease with which you could make an appointment |
| The time between making the appointment and seeing the provider |
| Telephone access to the provider |
| The personal attention you received from your provider |
| The extent to which the provider tries to understand your problem |
| The extent to which you shared in decision-making about your treatment |
| The degree of satisfaction with the final treatment decision |
| The extent to which you were informed about your condition and treatment |
| The extent to which you understood this information |
| Overall provider score |

| ***Quality of care Diabetes Mellitus Type 2 (patients enrolled into the care program)^**^*** |
| --- |
| Percentage of patients tested at least once for HbA1C levels in the past 12 months |
| The percentage of patients with HbA1C levels < 53 mmol/mol, of those tested |
| The percentage of patients with HbA1C levels > 69 mmol/mol, of those tested |
| Percentage of patients whose blood pressure was tested at least once in the past 12 months |
| The percentage of patients with a systolic blood pressure < 140 mm Hg, of those tested |
| Percentage of patients whose lipid profile was measured in the past 12 months |
| The percentage of patients with LDL-cholesterol levels < 2,5 mmol/l, of those tested |
| Percentage of patients whose renal clearance was determined at least once in the past 12 months |
| Percentage of patients whose albumine clearance was tested at least once in the past 12 months |
| Percentage of patients who have received an eye examination at least once in the past 24 months |
| Percentage of patients with diabetic retinopathy, of those tested |
| Percentage of patients who have received a foot examination at least once in the past 12 months |
| Percentage of patients whose smoking behavior was registered |
| Percentage of patients smoking, of those for whom smoking behavior was registered |
| Percentage of patients whose BMI was determined at least once in the past 12 months |
| Percentage of patients with a BMI < 25 kg/m^2^, of those tested |

| ***Quality of care COPD (patients enrolled into the care program)^**^*** |
| --- |
| Percentage of patients whose smoking behavior was registered |
| Percentage of patients who smoke, out of those for whom smoking behavior was registered |
| Percentage of patients whose level of physical activity was registered |
| Percentage of patients who had their inhalation technique tested at least once in the past 12 months |
| Percentage of patients who received spirometry based on FEV1/FVC ratio post BD in the past 12 months |
| Percentage of patients who have received a flu shot |
| Percentage of patients who experienced an exacerbation at least twice in the past 12 months |

| ***Quality of pharmacy care^***^*** |
| --- |
| Percentage of users of who use metformine as an anti-diabetic drug |
| Percentage of users of statines whose last receipt contained simvastatine, out of those who use lipid lowering medication |
| Percentage of users of triptans, whose dosage is less than 72 tables sumatriptan or less than 48 tables of other triptans |
| Percentage of new users of non-steroidal anti-inflammatory drugs, who use ibuprofen, naproxen or diclofenac |
| Percantage of users of simvastatine, who receive dosages of 40mg |
| Percentage of chronic users (40-79 years) of nitrates or platelet aggregation inhibitors who have received statins |
| Users of angiotensin II receptor antagonists, who use the generic product (losartan, valsartan, etc.) |

| ***Structure******* |
| --- |
| The NHG practice accreditation program |

* As developed by the participating providers (names available upon request)

** As developed by the ‘Landelijke Organisatie voor Ketenzorg’ [1]

*** As developed by the ‘Instituut voor Verantwoord Medicijngebruik’ [2]

**** As defined by the ‘Nederlands Huisartsengenootschap’ (issuer) [3]

**References**

1. Transparante ketenzorg Diabetes Mellitus, COPD en VRM Rapportage zorggroepen over 2012 [database on the Internet]2013. Accessed:

2. Instituut voor Verantwoord Medicijngebruik. Indicatoren voor de Monitor Voorschrijfgedrag Huisartsen2014.

3. Nederlands Huisartsen Genootschap. 2015. <http://www.nhg.com>.
